# Microinjection of specific anti-IMPDH2 antibodies induces disassembly of cytoplasmic rods/rings that are primarily stationary and stable structures

**DOI:** 10.1186/2045-3701-5-1

**Published:** 2015-01-05

**Authors:** Gerson Dierley Keppeke, Luís Eduardo C Andrade, Scott S Grieshaber, Edward K L Chan

**Affiliations:** Department of Oral Biology, University of Florida, 1395 Center Drive, Gainesville, FL 32610-0424 USA; Rheumatology Division, Universidade Federal de São Paulo, Rua Botucatu 740, São Paulo, SP 04023-062 Brazil; Immunology Division, Fleury Medicine and Health Laboratories, Avenida Gal Waldomiro Lima 508, São Paulo, SP 04102-050 Brazil; Department of Biological Sciences, University of Idaho, 875 Center Drive, Moscow, ID 83844 USA

**Keywords:** CTP synthase, Cytoophidium, Inosine monophosphate dehydrogenase, Intracellular compartment, Mycophenolic acid, Ribavirin

## Abstract

**Background:**

Our laboratory previously reported interesting rods 3–10 μm long and rings 2–5 μm diameter (RR) in the cytoplasm of mammalian cells. Experimental evidence show that both inosine-5'-monophosphate dehydrogenase 2 (IMPDH2) and cytidine triphosphate synthetase (CTPS) are components of RR structures. Several cell types, including mouse embryonic stem cells, and cell lines, such as mouse 3 T3 and rat NRK, naturally present RR structures, while other cells can present RR when treated with compounds interfering with GTP/CTP biosynthetic pathways. In this study, we aimed to investigate the dynamic behavior of these RR in live cells.

**Results:**

RR were detected in >90% of COS-7 and HeLa cells treated with 1 mM ribavirin or 6-Diazo-5-oxo-L-norleucine (DON) for 24 h, and in 75% of COS-7 cells treated with 1 mM mycophenolic acid (MPA) for the same period of time. Microinjection of affinity-purified anti-IMPDH2 antibodies in live COS-7 cells treated with ribavirin, DON, or MPA showed mature forms of RR presented as stable and stationary structures in 71% of cells. In the remaining 29% of cells, RR acquired erratic movement and progressively disassembled into fragments and disappeared within 10 min. The specific stationary state and antibody-dependent disassembling of RR structures was independently confirmed in COS-7 and HeLa cells transfected with GFP-tagged IMPDH2.

**Conclusions:**

This is the first demonstration of disassembly of RR structures upon microinjection of anti-IMPDH2 antibodies that led to the disappearance of the molecular aggregates. The disassembly of RR after microinjection of anti-IMPDH2 antibody further strengthens the notion that IMPDH2 are major building blocks of RR. Using two independent methods, this study demonstrated that the induced RR are primarily stationary structures in live cells and that IMPDH2 is a key component of RR.

**Electronic supplementary material:**

The online version of this article (doi:10.1186/2045-3701-5-1) contains supplementary material, which is available to authorized users.

## Introduction

Unique intracellular rods 3–10 μm long and rings 2–5 μm diameter (RR) have been characterized in the cytoplasm of many cell types [1–4]. Experimental evidence indicates that inosine-5'-monophosphate dehydrogenase 2 (IMPDH2) is a major component of the RR structures, but cytidine triphosphate synthetase (CTPS), another enzyme involved in nucleotide metabolism, has also been shown to be part of RR structures [2, 4, 5]. A recent review article has highlighted that many metabolic enzymes aggregate and form similar large fibers or intracellular bodies across the diverse organisms [6]. Although it is not yet clear whether all aggregates represent functional entities or storage bodies, examples of assembled fibers are discussed as a result of pathological damage in the enzymes (e.g. sickle-cell hemoglobin), enhance enzymatic activity (e.g. acetyl-CoA carboxylase), structural elements (e.g. actin fibers and microtubules), and to provide storage catalytic potential (e.g. CTPS) [6]. Three very recent reports show apparently contradicting conclusions regarding the enzymatic state of the CTPS enzyme when presented in these filamentary RR form. Barry et al. [7] concluded that the formation of RR in *E. coli* inhibits the activity of the CTPS enzyme and Aughey et al. [8] found similarly inhibited CTPS activity in RR in Drosophila tissues. However, Strochlic et al. [9] demonstrated that the CTPS within the RR structures in Drosophila are catalytically active. Thus the current hypotheses are that the assembly and disassembly of RR represent highly sensitive control of enzymatic activities by keeping enzymes in active/inactive forms and this can be an important mechanism of regulation of the indispensable GTP/CTP synthesis pathway within the cell. It should be noted that CTPS and IMPDH are rate-limited enzyme in de novo cytosine and guanine nucleotide biosynthesis, respectively.

Several cell lines naturally present RR structures including mouse 3 T3, rat NRK, and rat kangoroo Ptk2, as well as mouse embryonic stem cells [1], but for many other cancer cell lines grown *in vitro*, RR structures are usually not detected under normal culture conditions. However, in all cell lines treated to date with compounds interfering with GTP and CTP biosynthetic pathways, abundant RR structures are readily detected within minutes to hours [1]. For example, ribavirin and DON that inhibit IMPDH2 and CTPS, respectively, are highly capable of inducing RR structures that are readily observed with indirect immunofluorescence using antibodies to IMPDH2 or antibodies from patients with chronic hepatitis C viral (HCV) infection under treatment with ribavirin and interferon-α [2, 10–14]. In fact, it has been shown that HCV patients treated with IFN-α and ribavirin develop autoantibodies against RR after the sixth month of treatment, with titers increasing throughout treatment, and eventually decreasing after treatment interruption [11]. These autoantibodies seem to be, in most cases, directed against IMPDH2, which intriguingly is the prime target of ribavirin used in anti-HCV treatment in these patients [2, 10, 15].

The study of live cells has been critical for the elucidation of many cellular functions as well as dynamic interactions between organelles and other cell domains. An emblematic example is the characterization of intracellular cargo transport on microtubules. Live cell observations of microtubule vesicle transport revealed regulation of the polarity of trafficking and the roles of kinesins and dynein in the transport of cargo as well as a better understanding of the processes that regulate the behavior of transport at microtubule intersections [16–19]. Transfection of genes with fluorescent tags is a common method to label proteins for live cell investigation [20, 21]. This technique allows the study of the dynamic movement of a given protein or subcellular structure. This approach facilitates the real-time longitudinal observation of the target antigens in live cells subjected to a diverse array of *in vitro* conditions [22]. Transfection of an IMPDH2-GFP fusion construct, by Thomas et al. [3], to examine the aggregation of IMPDH2 into RR structures found diffuse cytoplasmic distribution of spicules that, by end-to-end or side-by-side fusions, clustered into macrostructures; for these experiments, only low expressing IMPDH2-GFP transfected cells were first sorted and examined as apparently high expressing cells failed to form RR-like structures. The latter is consistent with the report of Carcamo et al. [2] that high expression of IMPDH2-GFP in transfected cells inhibited the formation of RR structures, even when treated with ribavirin. Thus the expression levels of IMPDH2 affects the assembly of RR and this is largely consistent with the above discussion that linking these structures to the functional levels of the associated enzymes.

Another approach to study the behavior of biomolecules in live cells is the microinjection of fluorescent-labeled antibodies. The study of cellular structures in the presence of antibodies targeting their protein components can provide important information about intrinsic characteristics of the structure of interest [23–25]. For example, the microinjection of monoclonal antibodies to intermediate filaments into fibroblast cell lines causes them to break down into numerous small spheroid aggregates scattered throughout the cytoplasm [26, 27]. In fact, microinjection of antibodies against different cytoskeletal proteins was a fundamental approach in unveiling the ultrastructure, intermolecular relationships, and several functional aspects of important cell structures, especially intermediate filaments, in different tissues and cell lines.

The recently reported cytoplasmic RR structures are still poorly characterized. In this study, we aimed to investigate the spatial relationships of the RR structures over time in live cells as well as the behavior of these structures by antibody microinjection analysis.

## Results

**Figure 1:**
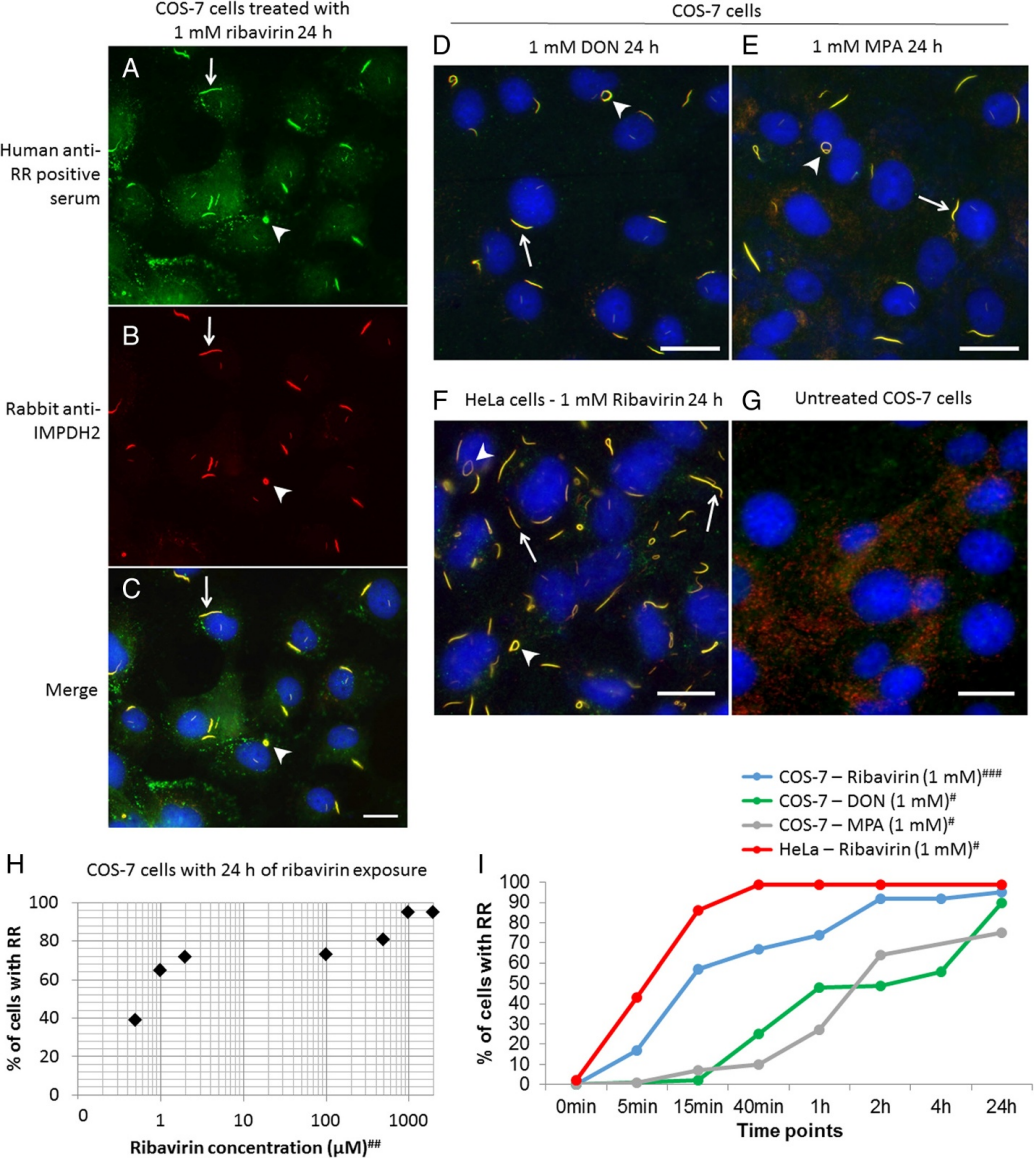
**Induction of rods and rings (RR) formation in COS-7 and HeLa cells with different inhibitors of the CTP/GTP biosynthetic pathway. (A-C)** RR structures induced in COS-7 cells by 1 mM ribavirin and detected by the prototype human anti-RR serum **(A)** and rabbit anti-IMPDH2 antibody **(B)**, with co-staining demonstrated in the merged image **(C)**. **(D-E)** Induction of RR with DON **(D)** and MPA **(E)** in COS-7. **(F)** HeLa cells treated with 1 mM ribavirin. **(G)** In untreated COS-7 cells, no RR was observed. **D**, **E**, **F**, and **G** show merged images of human anti-RR serum and rabbit anti-IMPDH2 staining. Yellow color in all merged images **(C-F)** means labeling by both probes. Nuclei were counterstained with DAPI (blue) in all images **(C-G)**. Bars: 20 μm. Arrows: rods; arrowheads: rings. **(H-I)** Quantitative analysis of RR induction. Different concentrations and time points of ribavirin showed 1 mM (1000 μM) and 2 h with >90% of RR in COS-7 cells. **(I)** DON and MPA showed in 2–4 h of treatment >50% of COS-7 with RR. **(H-I)** Experiment was repeated ^#^one, ^##^two or ^###^three times and ~130-150 cells were counted for each experiment.

Since COS-7 cells have been used extensively for live-cell imaging techniques, including microinjection, it was practical to adapt this system to analyze RR function. The first experiment was to validate if ribavirin-treated COS-7 cells were capable of RR formation. Human prototype anti-RR serum and rabbit polyclonal anti-IMPDH2 antibody were shown to recognize the characteristic set of RR structures in ribavirin-treated COS-7 cells (Figure 1A-C). The same was true in COS-7 cells treated with DON (Figure 1D) or MPA (Figure 1E). However, RR were not detected in untreated COS-7 cells as expected (Figure 1G). When the effect of the concentration of ribavirin was examined, the percentage of COS-7 cells presenting RR structures increased with the concentration of the drug. At 0.5 μM ribavirin, 39% of cells presented RR, and at 1 and 2 mM, 95% of cells presented the RR structures (Figure 1H). Most treated COS-7 cells presented only one RR structure per cell (Figure 1A-E). In contrast, more than 80% of HeLa cells treated with 1 mM ribavirin for 24 h presented four or more RR structures (42 cells examined; Figure 1F).In order to design the optimal live-cell imaging experiment for RR using COS-7 cells, it was necessary to determine how long it would take for RR to appear after treatment with various RR-inducing compounds. Time-lag experiments in COS-7 cells over a 24 h period were performed with 1 mM ribavirin, DON, or MPA, and RR structures were detected in 95%, 90%, and 75% of the treated cells, respectively (Figure 1I). About 50% of COS-7 cells were observed with RR structures after 15 min of ribavirin treatment. The induction of RR assembly with 1 mM DON or MPA was slower than that of 1 mM ribavirin. In contrast, HeLa cells demonstrated faster kinetics compared to COS-7 cells in RR assembly induced by 1 mM ribavirin and only 40 min exposure was sufficient to induce RR structures in 100% of the cells (Figure 1I).

The initial strategy was to microinject fluorescent-tagged anti-IMPDH2 antibodies into live COS-7 cells in order to follow the dynamics of RR. The rabbit anti-IMPDH2 antibody used is highly specific because it was an antigen-affinity purified preparation and showed only a single 55 kDa IMPDH2 band in immunoprecipitation analysis [2, 28]. To demonstrate that the chemically conjugated anti-IMPDH2 antibodies remained functionally intact, direct immunofluorescence with Alexa 488-conjugated rabbit anti-IMPDH2 antibody was performed in COS-7 cells treated with 0.1 mM ribavirin for 24 h. Out of 171 cells analyzed, 73% showed RR structures, indicating that the Alexa 488-conjugated rabbit anti-IMPDH2 antibody appeared to retain sufficient reactivity for our intended experiment (Additional file 1: Figure S1). In addition, we further investigated whether the various experimental conditions required for the microinjection setting could affect the assembly and disassembly of RR structures. After 24 h of treatment with 0.1 mM ribavirin, COS-7 cells were incubated for 2 h with the microinjection buffer, Hank's medium, or DNA dye Draq5. In all these required microinjection conditions examined, the ribavirin-treated cells continued to show the RR structures comparable to cells not exposed to such reagents (Additional file 1: Figure S2).

**Figure 2:**
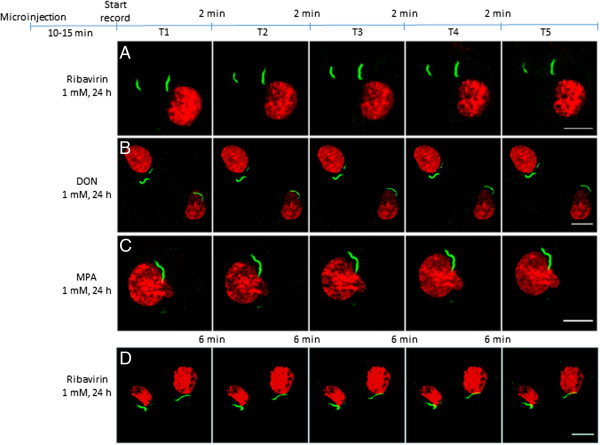
**RR structures induced by inhibitors of CTP/GTP synthesis in live COS-7 cells are in a stable and stationary state in ≈ 70% of the cells.** COS-7 cells were treated with ribavirin **(A)**, DON **(B)**, or MPA **(C)** for 24 h prior to microinjection with rabbit anti-IMPDH2 conjugated with Alexa 488 (green). After 10–15 min, sequential images (T1 to T5) were captured. Representative images from 2 min intervals for 10 min **(A-C)**, or 6 min intervals for 30 minutes **(D)**. No significant movement or relevant changes were observed in the size or shape of the cytoplasmic RR structures in most cells (71% out of 263 cells counted) during the observed time period of 10 min (n = 20) to 30 min (n = 5). Experiment was repeated independently 6 times in panel **(A)** and two times in panel **(B)** and **(C)**. Nuclei were stained with Draq5 (red). Bars: 10 μm.

**Figure 3:**
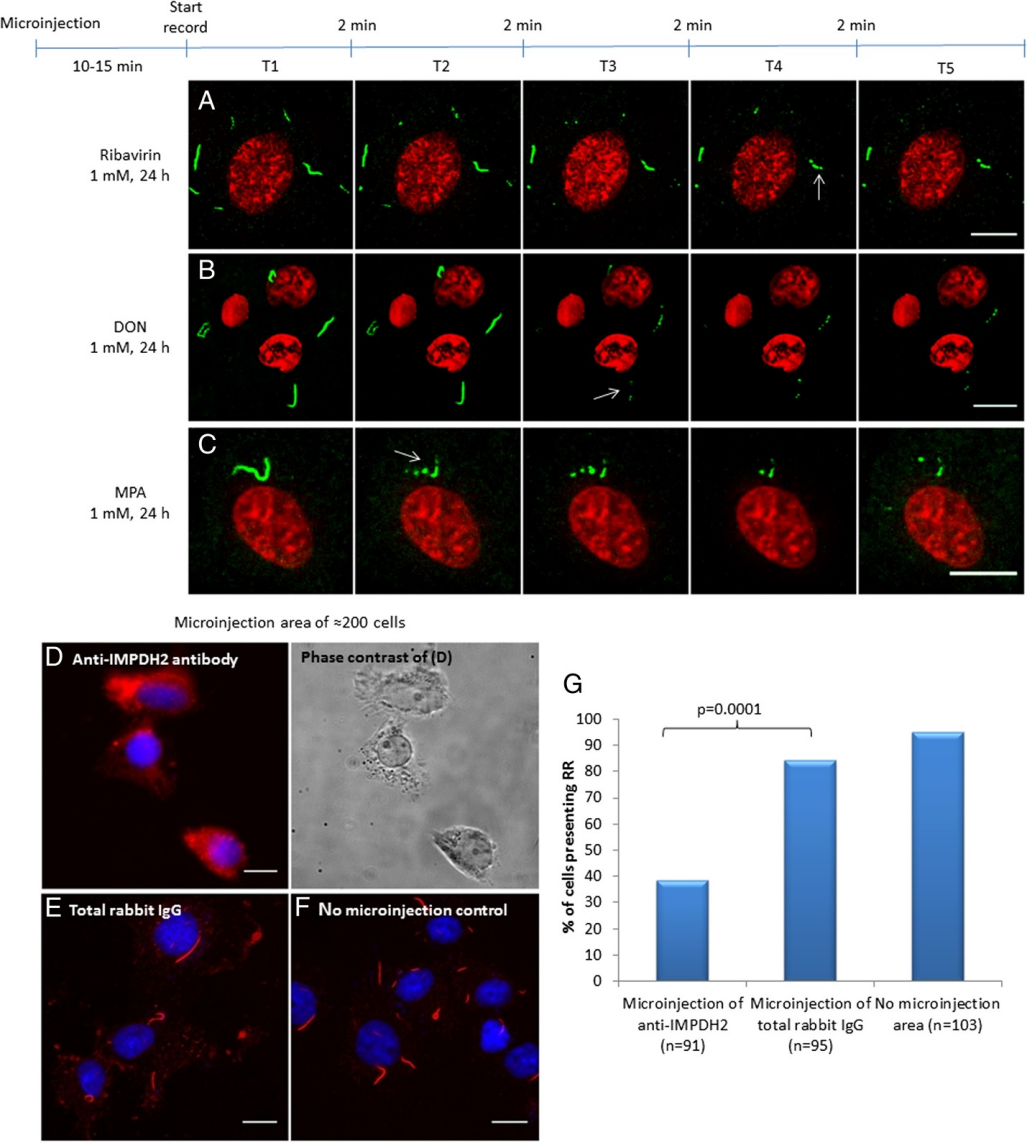
**RR structures induced by inhibitors of CTP/GTP synthesis disassembled in the remaining ≈ 30% of the cells by microinjection of anti-IMPDH2 antibody.** COS-7 cells were treated with 1 mM **(A)** ribavirin, **(B)** DON, or **(C)** MPA for 24 h and went under microinjection. Independent of the treatment used to induce RR, disassembly was detected in 76 (29%) of the 263 cells counted (check Figure 2). After 10–15 min of microinjection, images were captured in intervals of 2 min for up to 10 min to document the disassembly of RR. The arrows in A, B, and C indicate RR structures disassembling into pieces. Nuclei were stained with Draq5 (red). In **D**, **E**, and **F**, to evaluate the influence of the microinjection procedure on RR stability, anti-IMPDH2 antibodies or total rabbit IgG were microinjected into ~200 COS-7 cells; after 30 minutes, cells were fixed with 3% paraformaldehyde and stained with anti-IMPDH2 antibody. Control using total rabbit IgG shows no influence on RR structures. Blue color shows DNA labeled by DAPI. Bars, 10 μm. **(G)** Complete disassembly of RR in cells microinjected with anti-IMPDH2 compared to those microinjected with control total rabbit IgG or neighboring cells not subjected to microinjection. The proportion of cells that show intact RR was decreased in the slide area that we injected anti-IMPDH2 when compared with total rabbit IgG area (p = 0.0001 by Two-tailed Chi-square test).

Microinjection of Alexa 488-conjugated rabbit anti-IMPDH2 antibodies in COS-7 cells showed that drug-induced RR maintained as intact, stable, and stationary structures, i. e., there was no significant movement or changes in size and shape of RR in the majority of cells (71% out of 263 cells counted), however in the remaining 29% of the cells RR structures disassembled. These stable and stationary effects were observed in cells 10 min after microinjection regardless of the drug treatment used (1 mM ribavirin, DON, or MPA for 24 h); images were captured at 2 min intervals for an additional 10 min, indicating no change over time independent of the type of drug used (Figure 2). In most cases, the cytoplasmic RR structures did not show relevant changes in shape or size, even in video images captured at 10-second intervals for 10 minutes (Additional file 2: Movie S1). The same was observed when cells were followed for a longer time up to 30 min (Figure 2D). In 76 (29%) of the 263 COS-7 cells that received microinjection, RR structures were observed disassembling into fragments and eventually disappeared, independent of which drug was used to induce RR (Figure 3). Images were captured at 10-second intervals to observe the disassembly and erratic movements of ribavirin-induced RR (Additional file 3: Movie S2) and MPA-induced RR (Additional file 4: Movie S3). As a control, we compared the influence of microinjection of anti-IMPDH2 antibody versus total rabbit IgG in a slide area with ~200 COS-7 cells (Figure 3D, E and F). The fraction of cells presenting RR was much less in the area microinjected with anti-IMPDH2 antibody (38%; total = 91) compared to those in the same slide microinjected with control total rabbit IgG (84%; total = 95) (p = 0.0001 by Two-tailed Chi-square test). In the same slide, the fraction of cells without microinjection bearing RR was 95% (total =103, Figure 3G). To determine whether the disassembly of RR was dependent on the amount of anti-IMDPH2 antibody microinjected, COS-7 cells were microinjected for either 0.2 s each (~8,000 antibody molecules) or 2 s each (~80,000 antibody molecules). Disassembly of RR was observed in 13% of cells with 0.2-s microinjection versus 48% in cells with 2-s microinjection 20 min later (Additional file 1: Figure S3). This data suggest that the disassembly of some induced RR was mediated by the higher level of microinjected anti-IMPDH2 antibody in those cells (see below) while in cells microinjected with low level of antibody, the antibody served mainly to label RR and demonstrating their stationary state.

**Figure 4:**
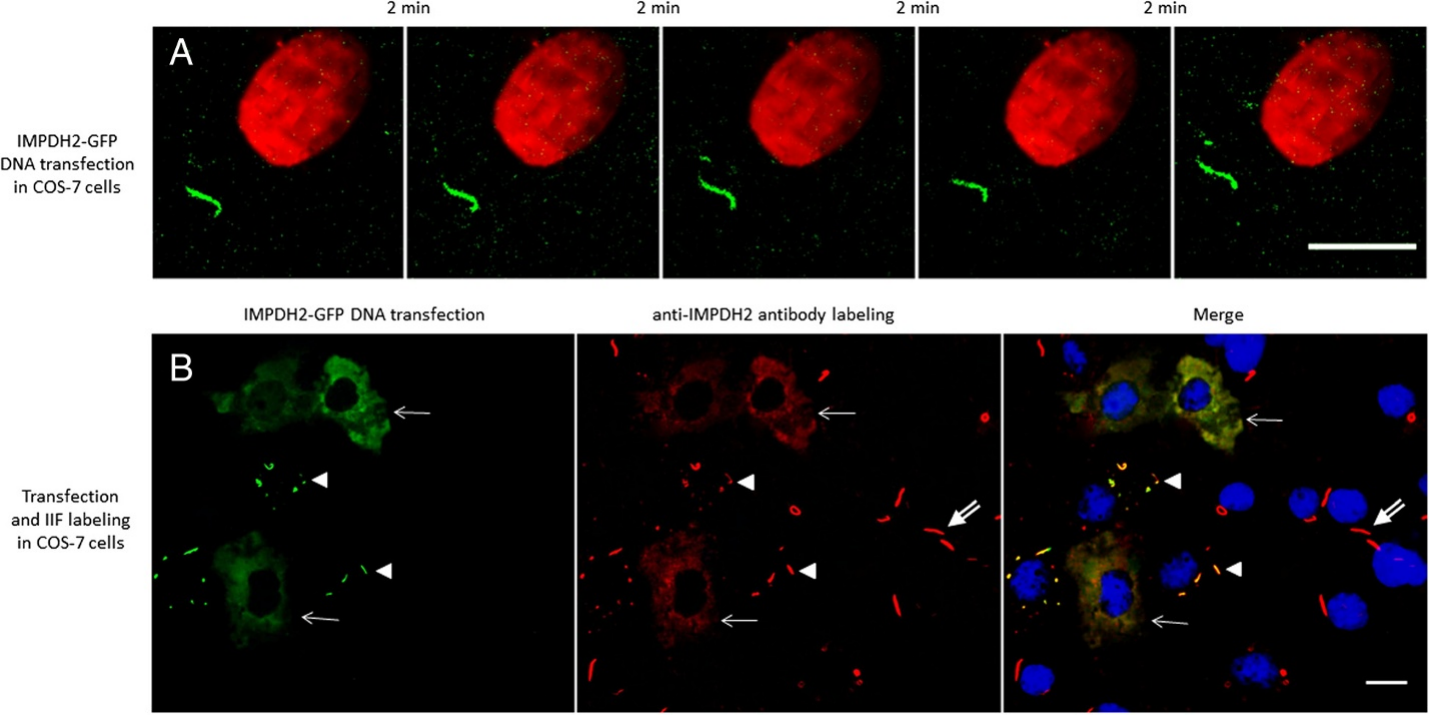
**IMPDH2-GFP-tagged RR induced in live COS-7 cells are detected in stable and stationary state.** COS-7 cells were transfected with IMPDH2-GFP plasmid and maintained in medium containing 1 mM ribavirin for 18 h. **(A)** Sequential pictures were captured for >10 min and representative images are shown at 2 min intervals. Similar to the antibody labeling experiment, changes in shape and size of RR were not observed. **(B)** Transfected cells were fixed and labeled by IIF with rabbit anti-IMPDH2 antibody (red). Cells with a high level of transfection (arrows, left panel) do not show RR (arrows). Cells with low transfection levels show RR (arrowheads). Of 336 cells counted, 16% show IMPDH2-GFP RR; transfection efficiency was 37%. Cells not transfected by IMPDH2-GFP showed RR labeled by anti-IMPDH2 antibody (double-arrows). All transfected IMPDH2-GFP-tagged RR (green, arrowheads) were labeled by rabbit anti-IMPDH2 antibody (red). Nuclei were counterstained with DAPI (blue). Data represent three in **(A)** and two in **(B)** independent experiments. Bars: 10 μm.

To obtain independent support for the results of the microinjection experiment above, transfection of an IMPDH2-GFP fusion construct was performed to rule out effects that might be from the interference of antibody microinjection. In transfected COS-7 cells treated with 1 mM ribavirin for 18 h, RR structures were also observed to be stable and stationary, showing no changes in shape and size within a 10 min interval (Figure 4A). The same was true when images were captured at 30 second intervals for 10 min (Additional file 5: Movie S4). In this same experiment, similarly transfected cells were also examined by IIF with anti-IMPDH2 (Figure 4B). Transfected cells (arrows and arrowheads, left panel) showed two distinct phenotypes. Interestingly, cells with very high transfection levels (arrows), gauged by high diffuse cytoplasmic GFP fluorescence (left panel), did not show RR structures, even when labeled by anti-IMPDH2 (arrows). The second type of transfected cells (arrowheads) with relatively low expression of IMPDH2-GFP showed distinct RR structures (left panel) that are also labeled by anti-IMPDH2 antibody (mid and right panels). No relevant diffuse cytoplasmic fluorescence is observed in these cells. Non-transfected cells show typical RR recognized by anti-IMPDH2 (double arrows).

COS-7 cells were used in the present study due to the increased thickness of these cells, which facilitates the procedures of microinjection. However, all experiments were also performed independently in HeLa cells to validate that the present observation was not limited to COS-7 cells. The follow-up for 10 min after microinjection of HeLa cells (24 h after treatment with 1 mM ribavirin) also showed stationary RR structures, with no changes in shape and size (Additional file 1: Figure S4A). No changes in size or shape of RR structures were observed in HeLa cells labeled by transfected IMPDH2-GFP (Additional file 1: Figure S4B), similar to COS-7 cells. The GFP-tagged RR structures in transfected HeLa cells, as in COS-7 cells, were also recognized by anti-IMPDH2 in IIF (arrow, Additional file 1: Figure S4C).

## Discussion

Our overall data showed that RR were stationary and stable structures in imaged live cells. This means cell maintained intact RR structure with no change in size and shape (stable) and showed no significant movement (stationary). During independent observation of live cells transfected with GFP-tagged IMPDH2, RR also appeared stationary and stable in size and shape.

In our previous study, RR were induced by ribavirin, MPA, and DON, that inhibit enzymes involved in the biosynthetic pathway of guanosine triphosphate (GTP) and cytidine triphosphate (CTP) [1, 2]. In this work, RR structures were examined by induction with ribavirin and MPA, which inhibited the IMPDH2 enzyme in an irreversible and reversible manner, respectively. RR structures induced by DON, which inhibits the enzyme CTPS in the CTP biosynthetic pathway, were also recognized by anti-IMPDH2 antibody. In experiments with COS-7 cells, ribavirin induced a higher percentage of cells to form RR and at a faster rate compared to those induced with MPA or DON at the same concentration. This observation was consistent with our reported in the induction of RR in HEp-2 cells [2]. In this study, under all conditions of RR induction, similar results were observed where most structures appeared stable and stationary (~70% of cells).

HeLa cells had a higher number of RR structures per cell than COS-7 cells (average ≥4 versus 1) and faster kinetics in RR assembly; in just 40 minutes of treatment with ribavirin, 100% of HeLa cells already presented RR, in contrast to 67% of COS-7 cells. This observation may be related to the amount of IMPDH2 enzyme available, which is influenced by the necessary IMPDH2 enzymatic activity for each cell line [29, 30]. The amount of IMPDH2 is also influenced by cellular nucleotide pools, which are related to cellular activities like mitosis and protein synthesis [31, 32]. We reported recently that glutamine deprivation for 72 h induced RR assembly in HeLa cells (purine and pyrimidine pathways are highly glutamine-dependent), and the RR structures disappeared within 15 min of adding 1 mM guanosine to the culture medium [33]. The higher frequency and faster kinetics of RR formation in HeLa compared to COS-7 cells could be based on other differences between these cell types, as COS-7 is originated from green monkey kidney while HeLa is from a human cervical cancer, and previous reports shows higher expression of IMPDH2 in cancer cells [34, 35]. Our data demonstrated that these cell types have different sensitivity to the induction of RR formation in the presence of different RR-inducing drugs. This in part might also be related to their difference in mitotic activity. In our experience, HeLa cells grow to confluence twice as fast as COS-7 cells.

Our study focused on the stationary and stable RR in live cells during the 30 min observation period. Other studies have examined the assembly process of RR. Gou et al. [36] explored the formation of cytoophidia (Greek for "cellular snakes", another name used to described RR-like structures) by CTPS-GFP transfection into mouse NIH/3 T3 cells treated with DON (50 μg/ml) for 10 min prior to recording live cell images. They showed small aggregates with dynamic movements forward and backward, and sometimes movement toward each other with end-to-end fusions to increase the length or side-by-side to increase thickness of the structures [36]. Another study by Thomas et al. [3] reported the clustering of GFP-tagged IMPDH2 in live HeLa cells treated with 2 μM MPA just before recording images and they also found diffuse cytoplasmic distribution of spicules that, by end-to-end or side-by-side fusions, clustered into macrostructures. In our experiments, the cells were exposed to the CTP/GTP pathway inhibitor drugs for much longer times, 24 h prior to anti-IMPDH2 microinjection and 18 h prior to recording in IMPDH2-GFP-transfected cells. The RR structures that we observed were already fully developed and mature, leading to the observed stationary and stable status. This explains why we did not capture any “pre-aggregation” changes (increase in thickness and length) in the short period of time that we recorded the structures; pre-aggregation changes in size and shape previously observed by others are probably related to the assembly process of RR.

The presence of the RR structure may explain some undescribed correlations between the loss of tolerance and autoantibody production in HCV patients treated with interferon/ribavirin [11, 12, 14, 28]. In fact, unpublished observations from our laboratory show that RR structures are present in the cytoplasm of peripheral blood mononuclear cells of HCV patients treated with ribavirin (manuscript in preparation). In this study, we observed that mature RR structures are primarily stable and stationary in live cells when followed for a short period of time. A limitation of this study, however, is that we only followed the cells for up to 30 min. Further studies are needed to determine why the IMPDH2 enzymes are clustering into RR under enzyme inhibition or nucleotide pool alterations. This will help to understand the function of cellular RR structures and why treated HCV patients produce anti-IMPDH2 autoantibodies.

We observed that microinjection of anti-IMPDH2 antibodies caused disassembling of RR structures in approximately 29% of COS-7 cells. Structures that disassemble in the presence of antibodies targeting themselves have been demonstrated before. Components of cytoskeletal intermediate filaments, in the presence of antibodies targeting specific structures, tend to be disassembled into cytoplasmic foci or clumps. For example, the microinjection of monoclonal antibodies to intermediate filaments into fibroblast cell lines caused these filaments to break down into numerous small spheroid aggregates scattered throughout the cytoplasm [26, 27, 37]. In a study of the Golgi complex, microinjection of anti-dynein heavy chain antibodies was shown to disperse the Golgi complex in about 50% of NRK cells, suggesting that the dynein motor is involved in establishing proper Golgi organization [38, 39]. The disassembling of RR structures in the presence of an anti-IMPDH2 antibody strongly indicates that IMPDH2 enzyme is an important element in RR, analogous to the demonstration of dynein as the motor in Golgi organization.

The disassembly of the RR structures after microinjection of anti-IMPDH2 antibody could be due to the steric hindrance generated by IMPDH2 epitope-paratope interaction, which potentially interferes with the aggregation or polymerization process of RR. In a parallel situation, the microinjection of antibodies targeting intermediate filaments, that cause them to break down into small spheroid aggregates, has been interpreted as the antibody physically blocking the region of the intermediate filaments that reacts with a cellular component which normally causes them to stretch out [37]. Antibody microinjection is a useful tool to study enzymatic activity because the antibody may block the enzyme activity *in vivo* [40]. In the case of RR structures, the IMPDH2 enzyme is already blocked by ribavirin and MPA treatment. In addition, the used anti-IMPDH2 antibodies are not known be able to block the enzyme activity.

An interesting point is that disassembly of RR structures was observed in only a fraction of the microinjected cells varying slightly from experiment to experiment. This may be due to the variability in the amount of the antibody microinjected, a methodological variable that is difficult to control completely [41, 42]. According to the Avogadro’s law (6.02 × 10^23^ molecules/mole), the number of antibody molecules microinjected into each cell was calculated to be 4,000 to 40,000 molecules, taking into consideration the time duration of microinjection in each cell (0.1 s ≈ 4,000 molecules). When COS-7 cells were microinjected for either 0.2 s each or 2 s each, the data supports a relationship that larger amount of antibody microinjected is correlated with the increase in the percent of cells showing disassembly of RR. This relationship allows for two possibilities within the cell: (1) a high level of injected antibody allows for increased binding to the RR structure, leading to a high rate of disassembly, or (2) low levels of injected antibody are not enough to significantly disturb the aggregation/polymerization process or potential activity of the RR structure, enabling us to follow the structure for a short time (10 to 30 min) without visualizing considerable alterations in shape or size.

In conclusion, we show for the first time the disassembly of RR structures in the presence of an antibody targeting IMPDH2, a molecular constituent of RR. By two independent methods, induced RR are demonstrated to be primarily stable and stationary structures in live cells. The disassembly of the RR structures in some cells after microinjection of anti-IMPDH2 antibody further strengthens the notion that IMPDH2 molecules are major building blocks of RR. Further studies exploring the chemical interaction among IMPDH2 molecules in the assembly of RR may provide a full understanding of how the RR structures are assembled in cell cytoplasm. However, it is acknowledged that our conclusion with induced RR, without further experiments, may not be generalized to naturally observed RR such as those in mouse embryonic stem cells, mouse 3 T3, or rat NRK cells.

## Materials and methods

### Cell culture and drug treatment

COS-7 cells from green monkey kidney and HeLa cells from human cervical cancer were obtained from the American Tissue Culture Collection (Manassas, VA) and cultured in 8-well Culture Slides (BD Falcon™; CA, USA) for transfection, drug treatment, and immunofluorescence (IIF) analyses. Alternatively, these cells were cultured on round 22 mm-diameter coverslips for microinjection procedures. Both cell lines were cultured in Dulbecco's Modified Eagle Medium (DMEM) with 10% Fetal Calf Serum (FCS) at 37°C and 5% CO_2_. Adherent cell lines were maintained at 50% confluence.

COS-7 and HeLa cells were treated with compounds reported to induce RR structures [2, 12]. Ribavirin (Sigma-Aldrich; R9644,) and mycophenolic acid (MPA, Sigma-Aldrich; M3536) were solubilized in tissue culture grade water to a 50 mM stock and 6-diazo-5-oxo-L-norleucine (DON, Sigma-Aldrich; D2141) was solubilized in water to a 100 mM stock and stored at -80°C until use. Drugs at various concentrations were added to cell culture 24 h prior to fixation.

### IIF procedure

Cells were fixed with 3% paraformaldehyde in PBS for 10 min, followed by permeabilization with 0.1% triton-X100/PBS for 5 min [43, 44]. For co-staining studies, cells were incubated for 1 h simultaneously with antigen-affinity purified rabbit anti-IMPDH2 polyclonal antibody (ProteinTech; 12948-1-AP) diluted 1/500 in PBS and human prototype anti-RR positive serum It2006 [2] also diluted 1/500 in PBS. Secondary antibodies were goat anti-rabbit IgG conjugated to Alexa Fluor® 568 (Molecular Probes; A-11036) and goat anti-human IgG conjugated to DyLight 488 (KLP- Kirkegaard & Perry Laboratories; 072-03-10-06), both diluted 1/500 in PBS. Secondary antibodies were also incubated simultaneously for 40 min. The entire IIF procedure was performed at room temperature in a wet chamber in the dark. Slides were sealed with VECTASHIELD® Mounting Medium with DAPI (VECTOR Labs, CA, USA) and analyzed at x400 magnification with fluorescence microscopy system (Carl Zeiss, Germany).

Rabbit anti-IMPDH2 antibody (0.26 μg/μL) was concentrated 5–6 fold using Amicon Ultrafilters (30-kDa cutoff, Millipore; UFC503008) according to the manufacturer’s recommended procedure. Labeling of the concentrated anti-IMPDH2 antibody was performed according to manufacturer recommendation using the Alexa Fluor® 488 Microscale Protein Labeling Kit (Molecular Probes; A-30006). The labeled antibody was resuspended in microinjection buffer (48 mM K_2_HPO_4_, 14 mM NaH_2_PO_4_, 4.5 mM KH_2_PO_4_, pH 7.2, sterilized by 0.22 μm filtering) and tested for activity in a direct fluorescence assay (diluted 1/300 and incubated 30 min) with COS-7 cells treated with 0.1 mM ribavirin for 24 h (Additional file 1: Figure S1). Despite the pre-labeling concentration process, the final concentration of labeled antibody determined by NanoDrop-1000 was 0.17 ng/nl.

### Microinjection of anti-IMPDH2

COS-7 and HeLa cells cultured on round coverslips were treated with ribavirin, DON, or MPA for 24 h to induce the formation of RR as previously described [2] prior to microinjection of anti-IMPDH2 antibody. Coverslips were transferred to the microscopy coverslips support (Leica DMIRB; Germany) with temperature control at 37°C, immersed in Hank’s medium (5.33 mM KCl, 0.44 mM KH_2_PO_4_, 138 mM NaCl, 4 mM NaHCO_3_, 0.3 mM Na_2_HPO_4_, 5.6 mM D-glucose, pH 7.2, sterilized by 0.22 μm filtering and added 1:100 antibiotics Penicillin 10,000 U/mL and Streptomycin 10,000 μg/mL). Microinjection of the antibody was performed by FemtoJet® System (Eppendorf). Femtotips needles (Type I, 1.0 μm, Eppendorf; 930000035) were loaded with 1-2 μL of either the Alexa Fluor 488-conjugated rabbit anti-IMPDH2 (0.17 ng/nL) or control rabbit total IgG. Microinjection was performed on average 4–6 microscopic fields at 1000× magnification (~100 cells). Pressure of injection was set at 2.0 to 3.0 psi and injection time between 0.1 to 1 second. Then, 3–5 cells were randomly chosen to be followed by confocal microscopy (Yokogawa QLC-100; AIC, New Jersey) and images captured with a camera (Cascade II: 512; Photometrics®), both attached to the microscope. Nuclei were visualized by staining with 5 μM Draq5 (BioStatus, United Kingdom; DR50050) added to the culture medium. Sliced images were captured for each chosen cell in 10–30 second intervals for 10 to 30 minutes.

### Transfection of IMPDH2-GFP

COS-7 and HeLa cells were seeded in 8-well chambers at 50–70% confluency (5 × 10^4^ cells/well) overnight prior to transfection. Cells were transfected with 0.24 ng/μL of IMPDH2-GFP DNA, cloned in the pCMV6-AC-GFP vector driven by the human cytomegalovirus (CMV) promoter (OriGene, Rockville, MD; RG202977), diluted in OPTi-MEM (Gibco, Cat.:31985–070), using Lipofectamine 2000 (Invitrogen, Cat.:11668–019) mixture at 1:1 ratio, and incubated for 6 h. After incubation, DMEM without antibiotics plus RR-inducing drug was added for 15–18 h. Transfection efficiency was 25-40%. The transfected cells were analyzed after fixation by IIF or directly as live cells using confocal microscopy. Sliced images were captured for randomly chosen cells in 30 second intervals for up to 10 minutes.

### Image analysis

Image sequences from confocal microscopy and IIF images were analyzed with OMERO.insight 4.4 and ImageJ 1.47 m with LOCI and Fiji plugins for Windows. Cells with or without RR structures were counted in captured images with ImageJ Analyze Particles tool, or by eye in randomly chosen microscopic fields. Statistical significance was determined with GraphPad Prism v5.0 using Two-tailed Chi-square test and p < 0.05 was considered significant.

## Electronic supplementary material

Additional file 1: Figure S1: Direct immunofluorescence of ribavirin-induced RR in COS-7 cells with rabbit anti-IMPDH2 conjugated with Alexa 488 (green). COS-7 cells treated with 0.1 mM ribavirin for 24 h were fixed and stained with Alexa 488-conjugated rabbit anti-IMPDH2 at 1:300 dilution for 30 min. Of 171 cells counted, 73% showed RR. Nuclei were counterstained by DAPI (blue). Bar: 20 μm. **Figure S2.** No observed changes in RR structures under various conditions used in our typical microinjection assay. COS-7 cells were treated with 0.1 mM ribavirin for 24 h (A) and followed by incubation in microinjection buffer (B), Hank’s medium (C), or Draq5 DNA dye (D) for 2 h. After 3% paraformaldehyde fixation, cells were stained with human anti-RR serum (green) and rabbit anti-IMPDH2 antibody (red). Nuclei were counterstained by DAPI (blue). Bar: 20 μm. **Figure S3.** Stationary RR structures detected in live HeLa cells. (A) HeLa cells treated with 1 mM ribavirin for 24 h were microinjected with Alexa 488-conjugated rabbit anti-IMPDH2 antibody. (B) HeLa cells transfected with IMPDH2-GFP were kept in medium containing 1 mM ribavirin for 18 h. Sequential pictures were captured from live cells and the images shown represent 2 min intervals for a total of 10 min. Nuclei were stained with Draq5 (red). (C) IMPDH2-GFP and anti-IMPDH2 antibody (red) labeled the same RR in transfected HeLa cells (arrows). Nuclei were counterstained with DAPI (blue). Bars: 10 μm. **Figure S4.** Dose-dependent effect of Alexa 488-conjugated anti-IMPDH2 antibody microinjected correlated with the level of RR disassembly. COS-7 cells were microinjected for 0.2 s each (n = 30) or 2 s each (n = 25) and followed for 20 min to observe the percent of cells demonstrating disassembly of RR. (PDF 603 KB)

Additional file 2: **Movie S1.** Ribavirin-induced RR structures in live COS-7 cells remain mostly stationary. COS-7 cells treated with 1 mM ribavirin for 24 h were microinjected with rabbit anti-IMPDH2 conjugated with Alexa 488 (green). After 10–15 min, sequential pictures were captured for 10 min at 10-second intervals. The video is a view of the pictures accelerated 100 times. Nucleus (red) was stained with Draq5. (AVI 2 MB)

Additional file 3: **Movie S2.** Disassembly of ribavirin-induced RR by the microinjection of anti-IMPDH2 antibody. COS-7 cells treated with 1 mM ribavirin for 24 h were microinjected with rabbit anti-IMPDH2 conjugated with Alexa 488 (green). After 10–15 min, sequential pictures were captured for 10 min at 10-second intervals. The video is a view of the pictures accelerated 100 times. RR structures are documented to disassemble into small fragments and some completely disappeared. Nucleus (red) was stained with Draq5. (AVI 1 MB)

Additional file 4: **Movie S3.** Disassembly of MPA-induced RR by the microinjection of anti-IMPDH2 antibody. COS-7 cells treated with 1 mM MPA for 24 h were microinjected with rabbit anti-IMPDH2 conjugated with Alexa 488. After 10–15 min, sequential pictures were captured for 10 min at 10-second intervals. The video is a view of the pictures accelerated 100 times. The rod was observed to break into pieces before complete disassembly. Nucleus (red) was stained with Draq5. (AVI 1 MB)

Additional file 5: **Movie S4.** RR in live COS-7 cells labeled by IMPDH2-GFP also show stationary behavior. After 18 h of IMPDH2-GFP transfection into COS-7 cells and 1 mM ribavirin treatment, sequential pictures were captured for 10 min with 30-second intervals. The video is a view of the pictures accelerated 100 times. No obvious changes in size or shape of RR structure (green) were observed. Nucleus (red) was stained with Draq5. (AVI 1 MB)

## Abbreviations

DAPI: 4',6-diamidino-2-phenylindole
DON: 6-Diazo-5-oxo-L-norleucine
EGFP: Enhanced green fluorescence protein
MPA: Mycophenolic acid
RR: Rods and rings.
