# Supplementary material for: Microinjection of specific anti-IMPDH2 antibodies induces disassembly of cytoplasmic rods/rings that are primarily stationary and stable structures
Source: Cell Biosci. 2015 Jan 5;5:1. doi: 10.1186/2045-3701-5-1 (PMC4298086; doi:10.1186/2045-3701-5-1)
Supplement: Supplementary file 1 — Additional file 1: Figure S1: Direct immunofluorescence of ribavirin-induced RR in COS-7 cells with rabbit anti-IMPDH2 conjugated with Alexa 488 (green). COS-7 cells treated with 0.1 mM ribavirin for 24 h were fixed and stained with Alexa 488-conjugated rabbit anti-IMPDH2 at 1:300 dilution for 30 min. Of 171 cells counted, 73% showed RR. Nuclei were counterstained by DAPI (blue). Bar: 20 μm. Figure S2. No observed changes in RR structures under various conditions used in our typical microinjection assay. COS-7 cells were treated with 0.1 mM ribavirin for 24 h (A) and followed by incubation in microinjection buffer (B), Hank’s medium (C), or Draq5 DNA dye (D) for 2 h. After 3% paraformaldehyde fixation, cells were stained with human anti-RR serum (green) and rabbit anti-IMPDH2 antibody (red). Nuclei were counterstained by DAPI (blue). Bar: 20 μm. Figure S3. Stationary RR structures detected in live HeLa cells. (A) HeLa cells treated with 1 mM ribavirin for 24 h were microinjected with Alexa 488-conjugated rabbit anti-IMPDH2 antibody. (B) HeLa cells transfected with IMPDH2-GFP were kept in medium containing 1 mM ribavirin for 18 h. Sequential pictures were captured from live cells and the images shown represent 2 min intervals for a total of 10 min. Nuclei were stained with Draq5 (red). (C) IMPDH2-GFP and anti-IMPDH2 antibody (red) labeled the same RR in transfected HeLa cells (arrows). Nuclei were counterstained with DAPI (blue). Bars: 10 μm. Figure S4. Dose-dependent effect of Alexa 488-conjugated anti-IMPDH2 antibody microinjected correlated with the level of RR disassembly. COS-7 cells were microinjected for 0.2 s each (n = 30) or 2 s each (n = 25) and followed for 20 min to observe the percent of cells demonstrating disassembly of RR. (PDF 603 KB) [file 13578_2014_198_MOESM1_ESM.pdf]

Additional file 1

Microinjection of specific anti-IMPDH2 antibodies induces disassembly of cytoplasmic rods/rings that are primarily stationary and stable structures

Gerson Dierley Keppeke, Luís Eduardo C. Andrade, Scott S. Grieshaber, and Edward K. L. Chan

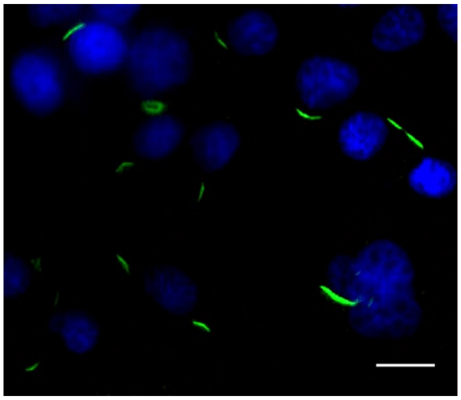

Figure S1. Direct immunofluorescence of ribavirin-induced RR in COS-7 cells with rabbit anti-IMPDH2 conjugated with Alexa 488 (green). COS-7 cells treated with 0.1 mM ribavirin for 24 h were fixed and stained with Alexa 488-conjugated rabbit anti-IMPDH2 at 1:300 dilution for 30 min. Of 171 cells counted, 73% showed RR. Nuclei were counterstained by DAPI (blue). Bar: 20 µm.

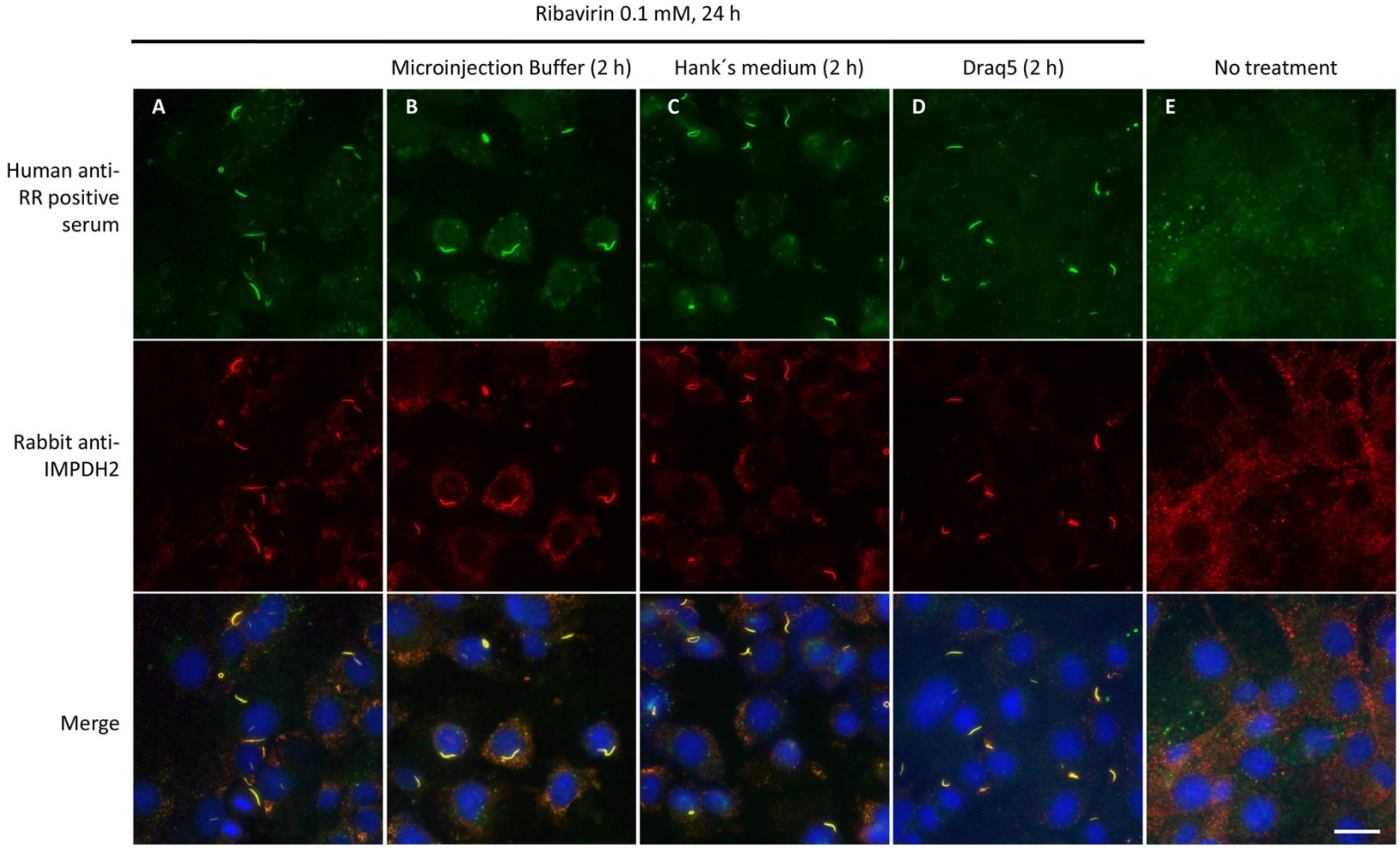

Figure S2. No observed changes in RR structures under various conditions used in our typical microinjection assay. COS-7 cells were treated with 0.1 mM ribavirin for 24 h (A) and followed by incubation in microinjection buffer (B), Hank's medium (C), or Draq5 DNA dye (D) for 2 h. After 3% paraformaldehyde fixation, cells were stained with human anti-RR serum (green) and rabbit anti-IMPDH2 antibody (red). Nuclei were counterstained by DAPI (blue). Bar: 20 µm.

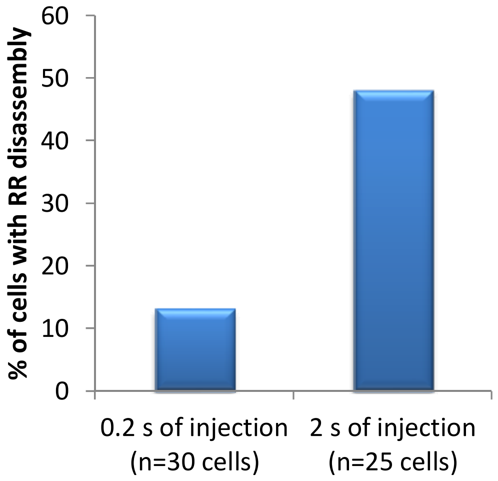

Figure S3. Dose-dependent effect of Alexa 488-conjugated anti-IMPDH2 antibody microinjected correlated with the level of RR disassembly. COS-7 cells were microinjected for 0.2 s each (n=30) or 2 s each (n=25) and followed for 20 min to observe the percent of cells demonstrating disassembly of RR. For the 0.2-s microinjections, 13% of cells showed RR disassembly and the level was elevated to 48% when the microinjection time was increased to 2 s.

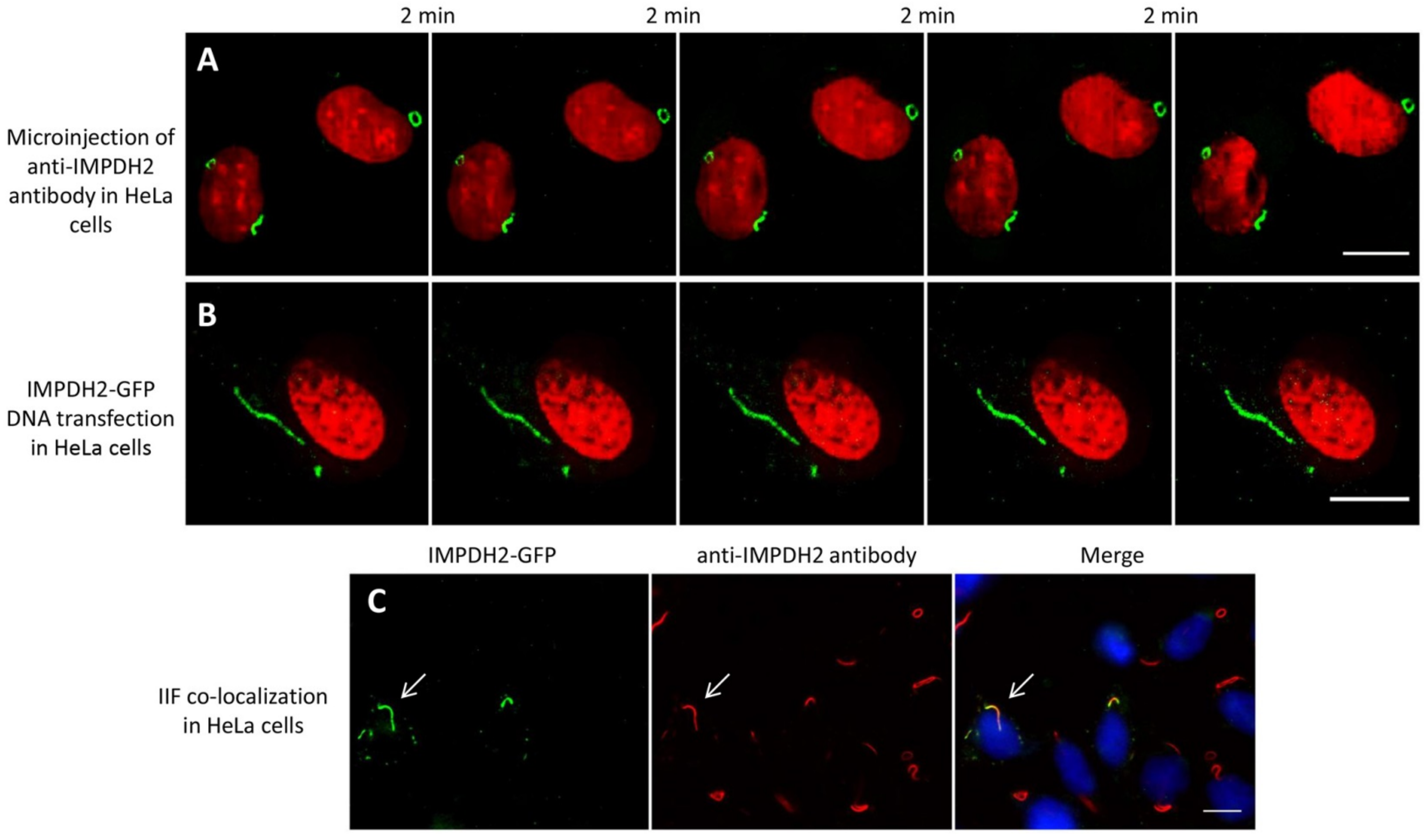

Figure S4. **Stationary RR structures detected in live HeLa cells.** (A) HeLa cells treated with 1 mM ribavirin for 24 h were microinjected with Alexa 488-conjugated rabbit anti-IMPDH2 antibody. (B) HeLa cells transfected with IMPDH2-GFP were kept in medium containing 1 mM ribavirin for 18 h. Sequential pictures were captured from live cells and the images shown represent 2 min intervals for a total of 10 min. Nuclei were stained with Draq5 (red). (C) IMPDH2-GFP and anti-IMPDH2 antibody (red) labeled the same RR in transfected HeLa cells (arrows). Nuclei were counterstained with DAPI (blue). Bars: 10 µm.
